# The influence of poverty and rabies knowledge on healthcare seeking behaviors and dog ownership, Cameroon

**DOI:** 10.1371/journal.pone.0197330

**Published:** 2018-06-21

**Authors:** Galileu Barbosa Costa, Amy Gilbert, Benjamin Monroe, Jesse Blanton, Sali Ngam Ngam, Sergio Recuenco, Ryan Wallace

**Affiliations:** 1 Poxvirus and Rabies Branch, Division of High-Consequence Pathogens and Pathology, Centers for Disease Control and Prevention, Atlanta, Georgia, United States of America; 2 ORISE Fellow, Poxvirus and Rabies Branch, Division of High-Consequence Pathogens and Pathology, Centers for Disease Control and Prevention, Atlanta, Georgia, United States of America; 3 Laboratório de Vírus, Departamento de Microbiologia, Instituto de Ciências Biológicas, Universidade Federal de Minas Gerais, Belo Horizonte, Minas Gerais, Brazil; 4 National Wildlife Research Center, US Department of Agriculture, Animal and Plant Health Inspection Service, Wildlife Services, Fort Collins, Colorado, United States of America; 5 Laboratoire National Vétérinaire (LANAVET), Garoua, Cameroon; 6 National Centre for Public Health, Instituto Nacional de Salud, Lima, Peru; Wistar Institute, UNITED STATES

## Abstract

**Background:**

Rabies is a fatal encephalitis caused by lyssaviruses, with most human cases worldwide resulting from rabid dog bites. Although effective animal and human vaccines have been available for over 100 years, control efforts have not been adequately implemented on the global scale and rabies remains one of the greatest global zoonotic threats to human health. We conducted a knowledge, attitudes and practices survey in Northern Cameroon to describe dog ownership characteristics, rates of dog bites, and post-bite healthcare seeking behaviors.

**Methods:**

The survey was performed in four rural Cameroonian communities. A structured community-based questionnaire was conducted over a 20-day period in April 2010, and focused on socio-economic factors correlated with gaps in rabies knowledge. Information pertaining to socio-demographics, as well as attitudes and practices with regard to animal bites and bite treatment practices were recorded. Characteristics of dog ownership such as dog confinement, resources provided to dogs, and dog vaccination status were examined. Human to dog ratios were compared on a linear scale to poverty scores by community. When applicable, 2-tailed Chi-square tests or Fisher’s exact tests were calculated to determine relationships between variables. We also used One-way Analysis of Variance (ANOVA) to identify associations between rabies knowledge and wealth with dog ownership, dog vaccination, and human healthcare seeking behaviors. Independent variables were evaluated using multivariate logistic regression analysis.

**Results:**

A total of 208 households were enrolled. Respondents were predominantly male (68.3%), with a median age of 43.6 years. Eighty-four households (39.9%) reported owning a total of 141 dogs (human dog ratio 10.4:1). The majority of dogs (61%) were allowed to roam freely. A history of rabies vaccination was reported for 30.8% of owned dogs. Respondents reported 11 bites during the two years preceding the survey (annual bite incidence was 2.6% [95% CI 1.4%– 4.6%]). Only one person (9.1%) received rabies post-exposure prophylaxis (PEP), and none described symptoms of clinical illness consistent with rabies. Respondents who indicated that they would seek medical care and PEP after a dog bite had higher average wealth and rabies knowledge index scores (p = 0.01 and 0.04, respectively). Respondents who indicated that they would seek care from a traditional healer had significantly lower wealth scores, but not significantly different knowledge scores (p < 0.01 and p = 0.49, respectively).

**Conclusions:**

In the communities evaluated, the majority of dogs were allowed to roam freely and had no history of rabies vaccination; factors that favor enzootic transmission of canine rabies virus. We also identified a strong relationship between poverty and dog ownership. Bite events were relatively common among respondents, and very few victims reported utilizing health services to treat wounds. Increased wealth and knowledge were significantly associated with increased likelihood that a respondent would seek medical care and post-exposure prophylaxis. These findings indicate the need for educational outreach to raise awareness of dog rabies and proper prevention measures.

## Background

Rabies is an acute encephalitis caused by the rabies virus, which is a single stranded negative sense RNA virus in the *Lyssavirus* genus (*Rhabdoviridae* family). The fatality rate of rabies approaches 100%, making it one of the most lethal of all infectious diseases [1,2]. Rabies is estimated to be responsible for 59,000 human deaths annually, in which >98% are attributed to bites from domestic dogs [2]. Most cases occur in low income countries [2,3]. The African continent accounts for 43% of the global rabies burden [2].

An estimated 200,000 persons receive post-exposure prophylaxis (PEP) for rabies each year in Africa. The number of annual human rabies deaths would be substantially higher in the absence of prophylaxis [1,2,4]. An estimated 76% of human rabies deaths in Africa occur in rural areas, and may result from a higher dog to human ratio, frequent lack of PEP, and/or reduced vaccination coverage in these areas as compared to urban settings [5]. The economic burden of human rabies in Africa is estimated to be $20 million per year, and the cost of PEP for the average person in Africa ($40) is likely to exceed their monthly gross income [2,5,6]. Major obstacles currently exist for assessing the risk of rabies in Africa. In general, lack of accurate data, financial investment, and adequate infrastructure are challenges that can contribute to inadequate disease surveillance [7]. To date, there has been inadequate surveillance of rabies throughout most of Africa, despite human rabies being notifiable in most countries [7–9]. It has been estimated that 95% of human rabies cases are likely unreported in eastern and southern Africa [9], again highlighting that the human rabies burden is substantially higher than currently recognized, making the estimation of rabies burden in Africa uncertain.

In the Central African nation of Cameroon, dogs are known to be the primary reservoir for rabies, but less is known about potential sylvatic endemic cycles [10,11]. The Laboratoire National Veterinaire (LANAVET) have reported more than 5,000 human rabies related exposures to animals of which 95% were related to dogs during the time period 2003–2008. Further evidence of the impact of rabies in Cameroon was reported by Awah-Ndukum et al reported 421 human rabies deaths and 330 laboratory confirmed rabid dogs during 1990–1999 [12]. Annual mass vaccination campaigns based on central-point methodology have been implemented in many parts of the country, supported by government officials since 2009 and are provided at a cost of 2 USD per dog vaccinated [10]. However, no post-vaccination monitoring to establish population immunity has been reported [10].

Enhanced surveillance activities to areas where human populations may be at the highest risk of rabies are recommended in order to assess risk and to evaluate effective strategies for control. Knowledge, attitudes, and practices (KAP) surveys are an useful way to assess owned dog population parameters, such as dog densities, animal bite injuries, and the health-seeking practices of the community with regard to animal bites injuries [13,14]. To better understand these factors in Cameroon, a KAP survey was conducted in four communities in northern Cameroon in areas of suspected high rabies incidence. In addition, we queried the population regarding control practices that may exist in these communities in response to suspected rabid animals.

## Methods

### Study design and population

**Fig 1:**
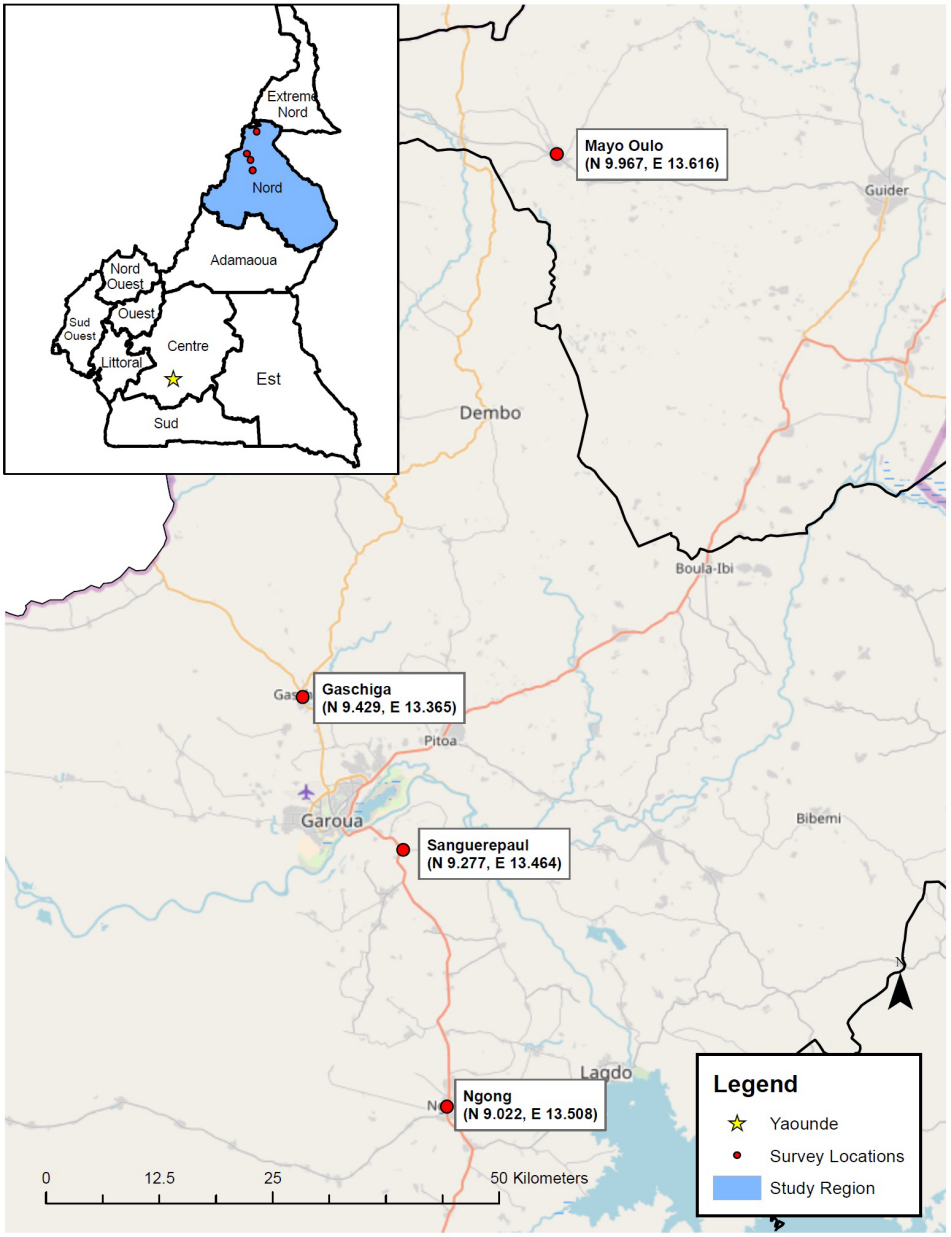
The study areas of northern Central African nation of Cameroon. The black outline identifies departments within the country. The Northern department, where the study was performed, is highlighted in blue in the inset. Locations of the surveyed communities are marked with red dots.

**Fig 2:**
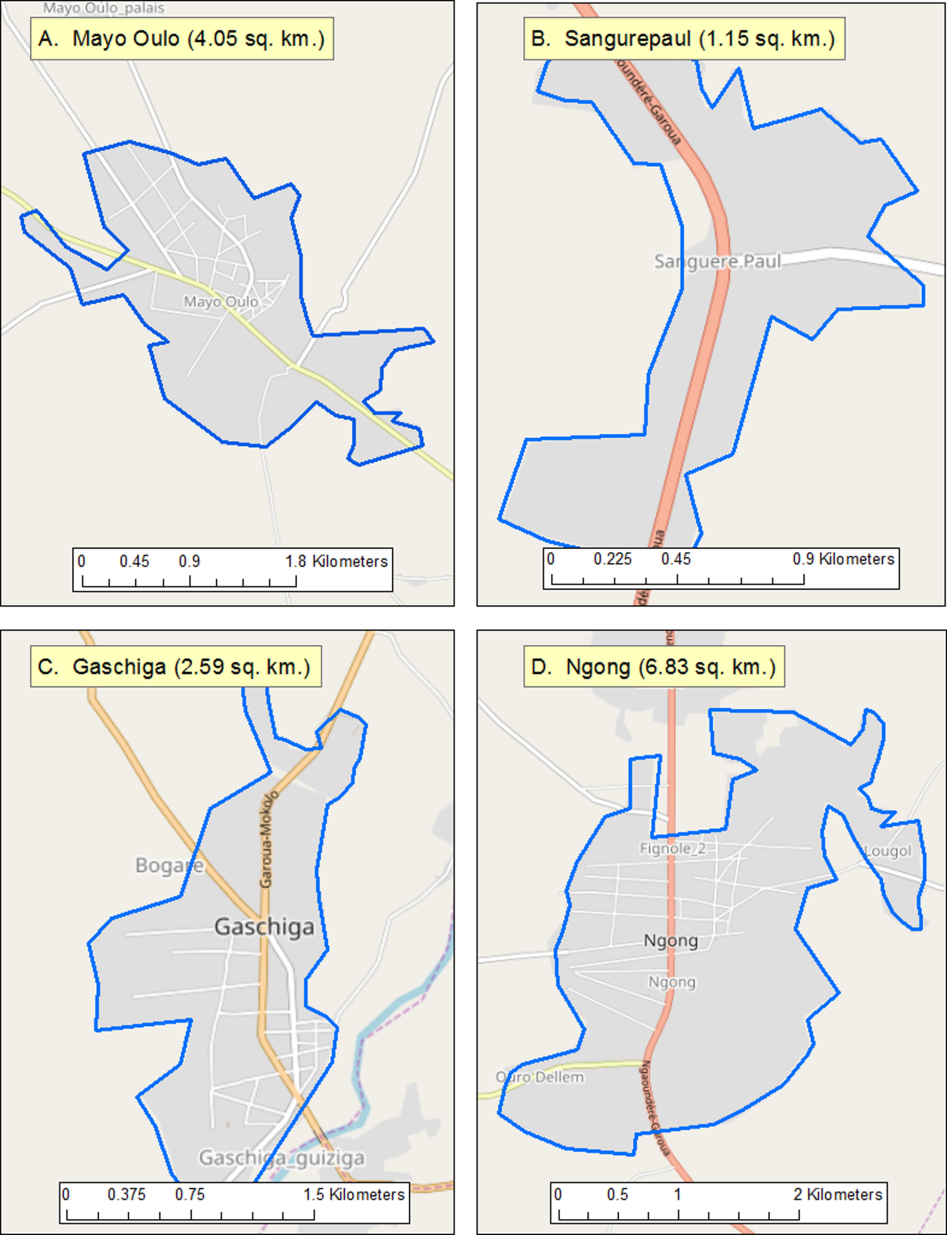
An open street map overview of imagery detailing the four communities surveyed in this study. Approximate population for the communities are: Mayo-Oulo 1,031 inhabitants, Sanguéré-Paul 377 inhabitants, Gaschiga 3,066 inhabitants, and Ngong 9,037 inhabitants.

Sanguéré-Paul Sanguéré-PaulA knowledge, attitudes and practices (KAP) survey was conducted in northern Cameroon, over a 20-day period in April 2010. Cameroon has an estimated population of 24,432,834 inhabitants, with 54.4% of residents located in urban areas (according to the CIA World Factbook [15]). Surveys were conducted in four communities in northern Cameroon: Mayo-Oulo, Gaschiga, Sanguéré-Paul, and Ngong (Figs 1 and 2). The communities surveyed constituted a convenience sample selected based on proximity to Garoua City, with qualifying communities being less than 4 hour drive. Households in individual communities were selected to represent a cross-section of income levels, i.e., presumed low, middle and high income, based on World Bank classifications of the income levels. All survey responses were recorded on handheld personal digital assistant (PDAs) devices, and GPS coordinates were taken for mapping purposes. Survey respondents consisted of consenting adult head of households (equal to or greater than 21 years of age). Respondents were not offered payment for their participation. Fingerprints or written informed consent was obtained for all respondents.

### Questionnaire

A structured questionnaire was developed in English, translated into French and reviewed by native Cameroonian people (S1 Appendix). The questionnaire consisted of 54 questions and was divided into four sections: socio-demographic, rabies knowledge, attitudes and practices towards animal bites, and bite treatment practices. The questionnaire was administered via face-to-face interviews, with responses entered in PDAs using GeoAge FAST software. Respondents were read questions, but were not provided answers from which to choose.

### Knowledge and wealth scores

A knowledge score was assigned following the model previously described by Tack et al. [16]. Four knowledge questions were assessed (Table 1). Each question had a maximum value of 10 points if correct responses were selected, and a minimum value of -10 points if only incorrect responses were selected. Positive and negative point values for individual responses were based upon the total number of possible responses (Table 1). The total values for each question were normalized, thereby negating the need to weight questions based on number of responses. When combined for a total knowledge score, these four equally weighted questions had a maximum of 40 points and a minimum of -40 points.

**Table 1:** Construction of the knowledge score. As indicated on the table below, all respondents were assigned a total of 10 points for correct answers in each of the 4 questions used to assess the knowledge about rabies. The knowledge questions were focused on: 1) severity of the disease; 2) transmission and reservoirs; and 3) attitudes regarding rabies exposure. For all questions, respondents were also deducted a total of -10 points for incorrect answers.

Questions	First preferred answer (points assigned)	Second preferred answer (points assigned)	Incorrect answers (points assigned)
1) How severe is the disease called rabies?^*^	Very severe (10 points)	NA	Mild (-10 points) Somewhat severe (-10 points)
2) How do humans get rabies from an infected animal?^**^	Bite (4 points) Contact with saliva (4 points)	Scratch (2 points)	Contact with blood (-2.5 points) Contact with urine or feces (-2.5 points) Observing the animal (-2.5 points) Touching the animal (-2.5 points)
3) What animals can be infected with rabies?^**^	Dogs (1.5 points) Bats (1.5 points) Jackals (1.5 points)	Cats (0.8 points) Livestock^†^ (0.8 points) Horses (0.8 points) Hyenas (0.8 points) Mongoose (0.8 points) Monkeys or other primates (0.8 points) Fox (0.8 points)	Poultry^†^ (-5 points) Wild birds (-5 points) I don’t know (0 points)
4) If you thought that you had an exposure to an animal with rabies, what would you do?^**^	Wash the wound (2.3 points) Actively seek medical treatment at a pharmacy, hospital, clinic or outpost (2.3 points) Receive rabies post-exposure prophylaxis (2.3 points)	Call a doctor (1.04 points) Confine the animal for observation (1.04 points) Submit the animal for disease testing (1.04 points)	Nothing (-10 points) Consult a traditional healer (0 points) Kill the animal (0 points)
Total points assigned	Maximum + 40 points		Minimum– 40 points

* Only one response allowed

** Multiple responses allowed

† Livestock includes cattle, sheep, and goats. Poultry includes chickens, ducks, and geese.

A wealth score was developed based on the socio-demographic variables collected: education level, house construction materials, and livestock (Table 2). Each variable had a maximum value of 10 points, distributed by high education level, high house building material quality, and maximum value for number of owned livestock. A minimum value of -10 points were attributed for illiteracy, lower house building material quality, and no owned livestock. The aggregate of these combined values was used to derive and estimated wealth score for the studied population. Descriptive parametric statistics such as mean scores, standard deviation, and 95% confidence intervals regarding knowledge and wealth scores were calculated. Wealth and knowledge scores were compared to the healthcare seeking behaviors.

**Table 2:** Education level, house construction, and livestock values used to construct the wealth score. The livestock values used to construct the wealth score are available at http://africafarming.info/how-much-does-an-animal-cost/.

Variables used to assess the wealth score		
**Years of schooling completed**	**Education level**	**Points assigned**
None	None	-10
≤ 6	Low	-3.3
7 to 12	Medium	+3.3
> 12	High	+10
Domicile feature*	**House building material quality**	Points assigned***
None**	Poor	-10
Soil, straw, palm fronds, curtain	Low	-3.3
Wood, brick, mud, sealed	Medium	+3.3
Cement, tile, metal, iron, glass	High	+10
**Livestock farming**	**Commercial value/head (in USD)**	**Points assigned**^**†**^
Cattle	$ 500,00	-10 ($ 0) -3.3 ($ 40,00–2.980,00) +3.3 ($ 3.100,00–5.400,00) +10 ($ 7.100,00–12.580,00)
Horses	$ 300,00	
Donkeys	$ 300,00	
Sheep	$ 120,00	
Goat	$ 80,00	
Chickens	$ 8,00	
Ducks	$ 8,00	

* For each house we evaluated the doors, windows, floor, walls and roof.

** Absence of doors and/or windows

*** There is no -10 points assigned for any household building material quality

† Points in livestock farming category were assigned based on the summary of all livestock owned

### Dog ownership

Characteristics of dog ownership, such as dog confinement, resources provided to dogs, vaccination status, and frequency of dog bites were examined. Respondents were asked to classify the confinement status of their dogs as: dogs which always stay at home, dogs which roam unsupervised at least part of the time or dogs which always roam unsupervised. Owners were asked about resources they provide to their dogs as well as resources provided to dogs they do not own, including food, water, shelter and veterinary care. When applicable, a 2-tailed Fisher’s exact test was calculated to determine the relationship between variables. Information on human household size and dog bite events in the past year were recorded to calculate the annual dog bite incidence among surveyed households. Wealth and knowledge scores were compared between dog owners and non-dog owners as well as owners of vaccinated dogs compared to owners of non-vaccinated dogs. Human to dog ratios were compared on a linear scale to poverty scores by community (n = 4). Correlations were calculated by the Pearson product-moment correlation coefficient (r), coefficient of determination (r^2^), and 1-tailed p-value (alpha = 0.05).

### Data analysis

For statistical analysis, categorical variables (age, sex, education level, household size, socioeconomic status, and bite action) were dichotomized (i.e., yes or no), and identified using a 2-tailed Fisher’s exact test with significance level of 5% (p ≤ 0.05) by using EPI-INFO software version 7.2 (http://www.cdc.gov/epiinfo/). The odds ratio and 95% confidence intervals were calculated when applicable. Extent of rabies knowledge and wealth score were analyzed for associations with attitudes and practices towards bite events via Student’s t test. We also used One-way Analysis of Variance (ANOVA) to compare differences for knowledge and wealth among communities. Values of p ≤ 0.05 were considered significant. To evaluate the independent association between the factors, a multivariate logistical regression model was applied, with p-values <0.1 considered to represent a significant association.

The total number of owned dogs reported by respondents was used as the denominator to obtain ratios of dogs per person and dogs per household. Furthermore, annual dog bite incidence was estimated using Person-Time Rate test (available in Open Epi software at http://www.openepi.com/), in which the bite event was the numerator and the total households the denominator. Vaccination coverage rates were assessed by the number of vaccinated dogs out of the number of owned dogs.

### Ethical considerations

The study was approved by the Centers for Disease Control and Prevention’s Human Research Office under the registration protocol #5891. All study participants were aged 21 years or older. An informed consent about the risks of participation in the study was provided from all participants. This data was collected on behalf a U.S. federal agency, and as such non-identifiable data is available upon request from the corresponding author with an appropriate data sharing agreement.

## Results

### Demographic profile of study population

Four rural districts were chosen for inclusion into this study: Mayo-Oulo, Gaschiga, Sanguéré-Paul, and Ngong (Figs 1 and 2). Two hundred-eight households were enrolled; one respondent per household—typically the head of household—was asked to complete the survey. The 208 households encompassed 1474 total persons (7.1 people per household). Demographic characteristics of the respondents are presented in Table 3. The median age of respondents was 43.6 years (ranging from 21 to 75 years). Men represented 68.3% of participants whereas women were 31.7%, and 64 individuals (30.9%) had completed ≤ 6 years of schooling. Within the communities, illiteracy rates were highest in Mayo-Oulo (60.0%).

**Table 3:** Characteristics of the 208 surveyed households within four communities surveyed, Cameroon, 2010.

Demographics	National average*	Community					
	Population	All households	Mayo-Oulo	Gaschiga	Sanguéré-Paul	Ngong	
	n = 24,360,803	n = 208 (%)	n = 66 (%)	n = 46 (%)	n = 26 (%)	n = 70 (%)	χ^2^
**Age (years)**							
21–30	18.5 years	46 (22.1)	14 (21.2)	10 (21.7)	7 (26.9)	15 (21.4)	0.69
31–40		45 (21.6)	13 (19.7)	11 (23.9)	7 (26.9)	14 (20.0)	
41–50		53 (25.5)	14 (21.2)	14 (30.4)	8 (30.8)	17 (24.3)	
51–60		30 (14.4)	9 (13.6)	5 (10.8)	2 (7.7)	14 (20.0)	
> 60		34 (16.4)	16 (24.2)	6 (13.0)	2 (7.7)	10 (14.3)	
**Gender**							
Male	50.1%	142 (68.3)	51 (77.3)	32 (69.6)	13 (50.0)	46 (65.7)	0.08
Female	49.9%	66 (31.7)	15 (22.7)	14 (30.4)	13 (50.0)	24 (34.3)	
Years of schooling completed*							
None	54.1%	97 (46.9)	39 (60.0)	18 (39.1)	8 (30.8)	32 (45.7)	**<0.01**^**†**^
≤ 6	21.4%	64 (30.9)	20 (30.8)	14 (30.4)	15 (57.7)	15 (21.4)	
7 to 12	15.3%	35 (16.9)	4 (6.1)	13 (28.3)	2 (7.7)	16 (22.9)	
> 12	9.2%	11 (5.3)	2 (3.1)	1 (2.2)	1 (3.8)	7 (10.0)	
**No. of people per household**							
1–3	7	10 (4.8)	1 (1.5)	5 (10.9)	1 (3.8)	3 (4.3)	**0.05**^**†**^
4–6		46 (22.1)	11 (16.7)	14 (30.4)	9 (34.6)	12 (17.1)	
> 6		152 (73.1)	54 (81.8)	27 (58.7)	16 (61.7)	55 (78.6)	
**Years lived in community**							
≤ 5	NA	21 (10.1)	8 (12.1)	3 (6.5)	5 (19.2)	5 (7.1)	0.44
6–10		25 (12.0)	7 (10.6)	4 (8.7)	2 (7.7)	12 (17.1)	
11–15		19 (9.1)	6 (9.1)	2 (4.4)	3 (11.5)	8 (11.4)	
16–20		36 (17.3)	9 (13.6)	7 (15.2)	5 (19.2)	15 (21.4)	
> 20		107 (51.4)	36 (54.6)	30 (65.2)	11 (42.3)	30 (42.9)	
**Wealth score**	29.3%^‡^	23.4	19.0	22.1	20.4	23.6	0.01^**^
		(22.2–24.5)	(17.3–20.7)	(19.5–24.7)	(17.7–23.1)	(21.5–25.7)	
**Knowledge score**	NA	15.8	15.1	14.8	15.6	17.2	0.23
		(14.8–16.8)	(13.4–16.9)	(12.6–16.9)	(13.3–17.9)	(15.6–18.9)	

* Cameroon national average for the presented variables were extracted from CIA factbook at: https://www.cia.gov/library/publications/the-world-factbook/geos/cm.html

† Exact p-value was estimated using Monte Carlo simulation.

‡ Average poverty level was measured as percent of people living below the international poverty line of US $1.90 per day

** Exact p-value was estimated using ANOVA.

*** poverty score was based on a scale of -20 to +20, with higher scores indicating a higher household net worth.

### Community dog-ownership and rabies vaccination status

Dogs were owned by 39.9% of the households. Survey respondents reported owning 146 dogs at the time the survey was conducted (1.8 dogs per dog-owning household) (Table 4). The dog ownership ratio, when considering the 1,474 persons represented in this survey was 10.1:1 (95% CI 8.6–12.6). Participants in Ngong community had the highest rate of dog ownership (6.8:1 humans to dogs) and individuals from Mayo-Oulo had the lowest (24.4:1 humans to dogs) (p < 0.0001).

**Table 4:** Demographics of dog-owning households in community members from Cameroon, 2010.

Demographics	All Households	Mayo-Oulo	Gaschiga	Sanguéré-Paul	Ngong
	N = 208 (%)*	N = 66 (%)*	N = 46 (%)v*	N = 26 (%)*	N = 70 (%)*
**Household Surveys Completed**	208	66	46	26	70
Owned Dogs**	141	20	29	16	76
**Dog-owning households**^**‡**^					
Yes	83 (39.9)	11 (16.7)	19 (41.3)	8 (30.7)	45 (64.3)
No	125 (60.1)	55 (83.3)	27 (58.7)	18 (69.2)	25 (35.7)
**Dogs per Dog Owning Household**	1.7	1.8	1.5	2.0	1.7
Total People***	1474	487	299	175	513
Human to dog ratio	10.4:1	24.4:1	10.3:1	10.9:1	6.8:1
**Dog-confinement status****†**					
Always home	55 (37.7)	10 (45.4)	3 (10.3)	5 (29.4)	37 (47.4)
Home and roam	89 (61.0)	12 (54.5)	26 (89.7)	12 (70.6)	39 (50.0)
Always roam	2 (1.4)	0	0	0	2 (2.6)
**Dogs vaccination status****†**					
All dogs vaccinated	37 (26.2)	11 (55.0)	2 (6.9)	0	24 (31.6)
Some dogs vaccinated	4 (2.8)	0	0	0	4 (5.3)
No dogs vaccinated	100 (71.0)	9 (45.0)	27 (93.1)	16 (100.0)	48 (63.1)
**Dog vaccination coverage**	30.8%	59.1%	6.9%	0	66.7%
**Poverty score**^**##**^	23.4	19.0	22.1	20.4	23.6
CI 95%	(22.2–24.5)	(17.3–20.7)	(19.5–24.7)	(17.7–23.1)	(21.5–25.7)
**Knowledge score**	15.8	15.1	14.8	15.6	17.2
CI 95%	(14.8–16.8)	(13.4–16.9)	(12.6–16.9)	(13.3–17.9)	(15.6–18.9)

* Percent in this category are indicated by column

** Owned dogs calculated from imputed survey values, rounded up to nearest whole number

*** Total people represented in the survey

‡ Exact p-value (< 0.0001) was estimated using Monte Carlo simulation

† Total of dogs in this category = 141

# linear regression analysis of poverty score and HDR showed high correlation with r^2^ = 0.76 and slope of -3.4 (p = 0.06)

## poverty score was based on a scale of -20 to +20, with higher scores indicating a higher household net worth.

Overall, there was a negative linear correlation between poverty and dog populations, with increased community poverty resulting in fewer owned dogs (r = -0.875, r^2^ = 0.76, slope = -3.4, p-value 0.062) (Table 2).

Only 1.4% of dog owners reported their dogs were allowed to roam freely at all times, whereas 61% of dogs were reported to roam freely part of the time, and 37.7% of dogs were reportedly always confined (Table 4). Eleven respondents indicated that they had been bitten by a dog in the past two years, representing an annual bite incidence of 2.6% (95% CI 1.4%– 4.6%). Gaschiga accounted for the highest bite incidence which was 4.3% (95% CI 1.3%– 11.1%), compared to the lowest observed in Sanguéré-Paul which was 0.55% (95% CI 0.15%– 1.6%). Forty-five dogs were reported to have received rabies vaccine at some point prior to the survey (vaccination coverage of 30.8%). The highest rates of rabies vaccination coverage was seen in Ngong (66.7%) and Mayo-Oulo (59.1%). Gaschiga had a coverage of 6.9%, whereas the lowest coverage was observed in Sanguéré-Paul (0%) (Table 4).

The majority of respondents reported that they provided their dogs with food (97.6%) and water (90.4%) (Table 5). However, only 8.4% provided any level of veterinary care to their dogs. Few dog owners (2.4%) reported that they do not provide their dogs with any of the resources assessed. In addition to care for owned dogs, 9.1% (n = 19) of respondents provided some form of care to dogs in the community that they did not own (community dogs); the most frequently reported resource was water (9.1%). No respondents reported that they provided veterinary care to community dogs.

**Table 5:** Care provided to owned and community dogs, Cameroon, 2010.

Type of care	Owned dogs		Community dogs	
	Total respondents = 83		Total respondents = 208	
	Number	%	Number	%
**Care combination**				
No care provided	2	2.4	189	90.9
Partial care provided	79	95.2	19	9.1
Full care provided	2	2.4	0	0
**Type of care provided**‡				
Food	81	97.6	13	6.3
Water	75	90.4	19	9.1
Shelter	4	4.8	0	0
Veterinary care	7	8.4	0	0
None	2	2.4	189	90.9

‡ Multiple responses allowed, totals may not add up to 100%

Two respondents who provided full care are also included in any care category

### Attitudes towards dog bite events and health seeking behaviors

Table 6 presents attitudes and practices towards bite events. When respondents were asked about their healthcare seeking behaviors if bitten by a dog they did not know/own, only 6% of respondents indicated that they would wash the wound, 36.7% indicated that they would call a doctor, and 52.6% reported that they would seek medical treatment. Only 3.1% of individuals indicated that they would seek PEP, and 9.7% would consult a traditional healer. Few respondents indicated that they would confine the biting dog for observation (2.6%), or would submit the dog for disease testing (8.7%). However, 23% of individuals indicated that would kill the dog. Furthermore, 92.8% of respondents (n = 193) reported that the main barrier for getting medical treatment in their community is the lack of money to pay for the treatment.

**Table 6:** Attitudes and practices towards bite events among community members, Cameroon, 2010.

Bite action	Bitten by a dog I do not know/own			
	Total participants N = 208*			
	Attitudes	Practices	Odds Ratio	p value^†^
	total respondents = 196	total respondents = 11	CI 95%	
	n (%)^**^	n (%)^**^		
**Wash the wound**				
Yes	12 (6.1)	2 (18.2)	3.4	0.2
No	184 (93.9)	9 (81.8)	(0.7–17.5)	
**Called a doctor**				
Yes	72 (36.7)	8 (72.7)	**4.6**	**0.02**
No	124 (63.3)	3 (27.3)	**(1.2–17.8)**	
**Actively sought medical treatment at a pharmacy, hospital, clinic or outpost**				
Yes	103 (52.6)	1 (9.1)	**0.09**	**0.004**
No	93 (47.4)	10 (90.9)	**(0.01–0.7)**	
**Received rabies PPE**				
Yes	6 (3.1)	1 (9.1)	3.2	0.4
No	190 (96.9)	10 (90.9)	(0.3–28.9)	
**Consulted a traditional healer**				
Yes	19 (9.7)	2 (18.2)	2.1	0.4
No	177 (90.3)	9 (81.8)	(0.4–10.3)	
**Confined the dog for observation**				
Yes	5 (2.6)	1 (9.1)	3.8	0.3
No	191 (97.4)	10 (90.9)	(0.5–35.8)	
**Submitted the dog for disease testing**				
Yes	16 (8.2)	0	0.5	0.8
No	180 (91.8)	11 (100.0)	(0.03–9.1)	
**Killed the dog**				
Yes	45 (23.0)	0	0.2	0.3
No	151 (77.0)	11 (100.0)	(0.009–2.6)	
**Respondents who did not indicate a healthcare seeking action**				
Yes	192 (98.0)	7 (63.6)	**0.03**	**0.0003**
No	4 (2.0)	4 (36.4)	**(0.007–0.18)**	

* Only one participant declined to answer this question

** Percentage is shown by column

† p value was calculated by using Fischer’s exact test

Among persons surveyed, 11 people were bitten by a dog in the past two years (2.6% annual bite incidence, 95% CI 1.4%– 4.6%). None of the bites were from animals known by the respondents. Among these 11 individuals 2 (18.2%) reported that they had washed the wound (Table 6). Eight individuals (72.7%) called a doctor and two (18.2%) individuals consulted a traditional healer. Only one person sought medical care and received PEP (9.1%). Dog confinement for observation was reported by only one individual. No respondents submitted the dog for disease testing or killed the dog. The reported rates of healthcare seeking behaviors for theoretical dog bites were compared to the practices of persons who truly experienced a dog bite. Bite victims were three times more likely to wash the wound than was reported under the theoretical bite scenario (95% CI 0.8–11.7). Bite victims were also twice as likely to call a doctor than was reported under the theoretical scenario (risk ratio 2.0, 95% CI 1.3–3.0, p = 0.02). Bite victims were 5.8 times less likely to seek medical care as compared to the theoretical scenario (95% CI 0.9–37.6, p < 0.01).

### Influence of knowledge and wealth status on community actions towards dog bite events and dog ownership

**Fig 3:**
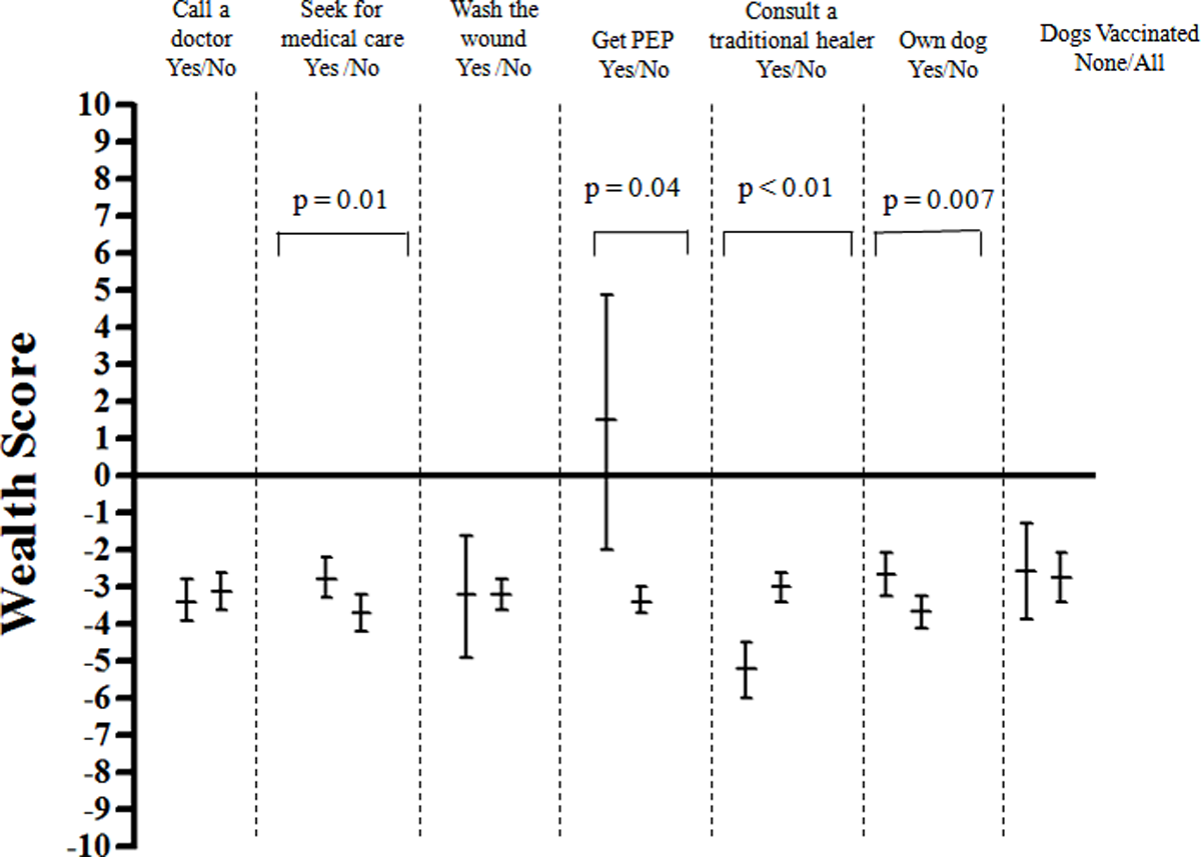
Analysis of wealth score and its association with practices towards bite events among community members, Cameroon, 2010. The horizontal midline of each vertical bar represents the mean index score; upper and lower confidence intervals are depicted respectively at either end. P-value was calculated by using T-test.

**Fig 4:**
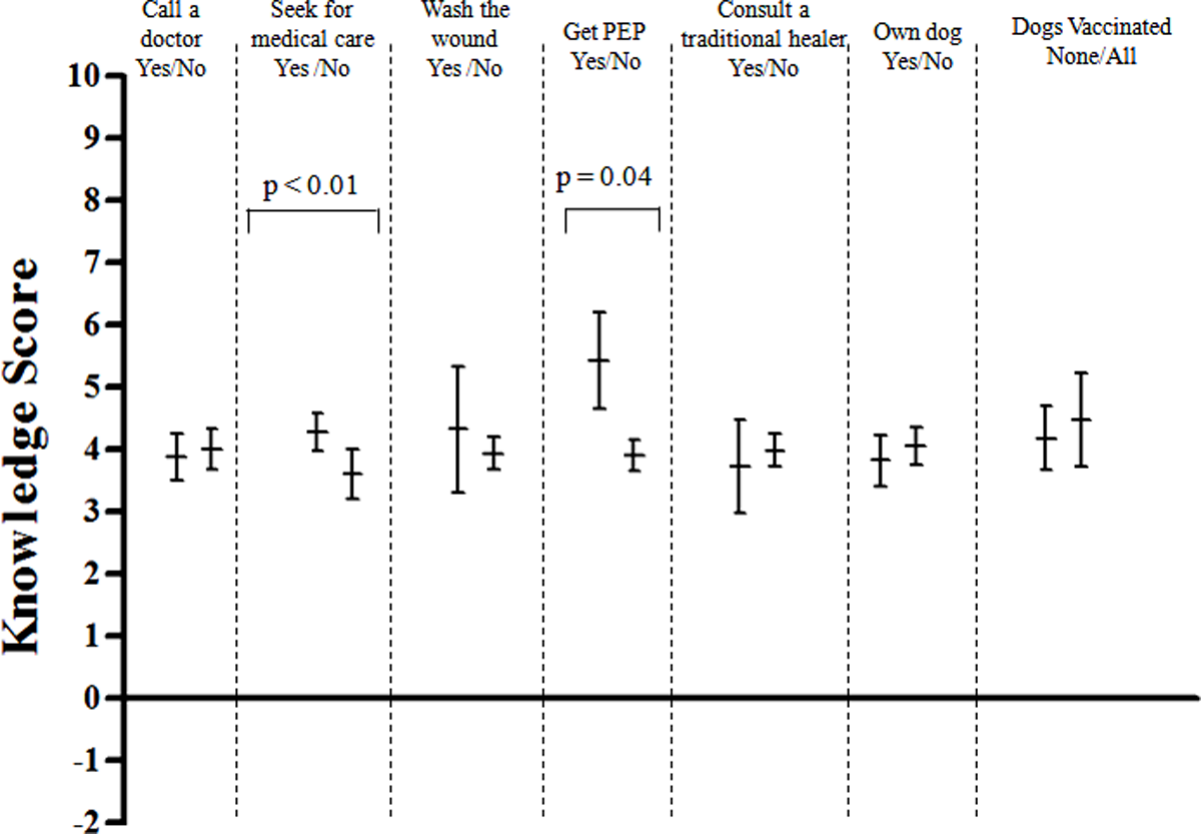
Analysis of knowledge score and its association with practices towards bite events among community members, Cameroon, 2010. The horizontal midline of each vertical bar represents the mean index score; upper and lower confidence intervals are depicted respectively at either end. P-value was calculated by using T-test.

Wealth scores were higher for those people who indicated that they would seek medical care for a bite compared to those who did not indicate they would seek medical care (averages = -2.8 and -3.7, p = 0.01) (Fig 3). Wealth scores were also higher for those individuals who indicated that they would seek PEP compared to those who did not indicate they would seek PEP (averages = 1.5 and -3.4, p = 0.04). Likewise, knowledge scores were higher for people who indicated that they would seek medical care for a bite compared to those who did not indicate they would seek medical care (averages = 4.3 and 3.6, p < 0.01) (Fig 4). Moreover, knowledge scores were higher for people who indicated that they would seek PEP compared to those who did not indicate they would seek PEP (averages = 5.4 and 3.9, p = 0.04).

Wealth scores were lower for those individuals who indicated that they would seek care from a traditional healer (averages = -5.2 and -3.0, p < 0.01). Knowledge scores were not different for persons who indicated they would seek care at a traditional healer compared to those who did not report this practice (averages = 3.7 and 4.0, p = 0.49). Wealthier households were more likely to own dogs (averages = -2.6 and -3.6, p = 0.007), however, dog ownership was not associated with rabies knowledge. Likewise, wealth and knowledge scores did not statistically differ for respondents with vaccinated dogs compared to unvaccinated dogs.

Variables independently associated with seeking medical care were knowledge score, poverty score, and gender (Table 7). Individuals with a higher knowledge score were more likely to seek medical care, whilst males and the poorest individuals were less likely to seek medical care. Alternatively, variables independently associated with consulting a traditional healer were knowledge score, age, and gender (Table 8). Individuals with a lower knowledge score were less likely to consult a traditional healer, whilst males and individuals older than 30 years were more likely to consult a traditional healer.

**Table 7:** Characteristics associated with individuals who actively sought medical care by multivariate logistic regression, Cameroon, 2010.

Variables	Sought medical care	Unadjusted OR	Adjusted OR**	p value
	(Yes/No)	(95% CI)	(90% CI)	
Knowledge*	15.8/max points	1.21 (1.0–1.4)	1.3 (1.1–1.5)	0.01
Poverty*	23.4/max points	0.9 (0.8–1.4)	1.2 (1.1–1.3)	0.003
**Age group (years)**				
21–30	25/20	1.03 (0.8–1.2)		
31–40	22/21			
41–50	24/26			
51–60	15/11			
> 60	14/10			
**Gender**				
Male	64/70	0.5 (0.2–0.9)	0.4 (0.3–0.9)	0.008
Female	39/23			
**Years of schooling completed**				
None	47/44	0.9 (0.8–1.1)		
≤ 6	28/31			
7–12	21/14			
> 12	7/3			
**No. of People per Household**				
1–3	5/4	1.14 (0.7–1.1)		
4–6	20/23			
> 6	78/66			
**Own dogs**				
Yes	41/38	0.5 (0.2–0.9)		
No	61/55			

* Knowledge and poverty were analyzed as continuous variables with average score values displayed in the table with maximum values as (average score/maximum score value)

** Adjusted odds ratios only displayed for variables that remained significant at alpha < 0.1

**Table 8:** Characteristics associated with individuals who consulted a traditional healer by multivariate logistic regression, Cameroon, 2010.

Variables	Consulted traditional healer	Unadjusted OR	Adjusted OR	p value
	(Yes/No)	(95% CI)	(90% CI)	
Knowledge and Poverty interaction*		1.3 (1.2–1.5)	1.3 (1.1–1.4)	0.002
**Age group (years)**				
21–30	0/45	1.03 (0.8–1.2)	1.8 (1.2–2.8)	0.02
31–40	1/42			
41–50	6/44			
51–60	3/23			
> 60	7/17			
**Gender**				
Male	17/117	0.5 (0.2–0.9)	15.4 (2.3–101.4)	0.01
Female	2/60			
**Years of schooling completed**				
None	18/73			
≤ 6	1/58	0.9 (0.8–1.1)		
7–12	0/35			
> 12	0/10			
**No. of People per Household**				
1–3	3/6	1.14 (0.7–1.1)		
4–6	5/38			
> 6	11/133			
**Own dogs**				
Yes	4/75	0.5 (0.2–0.9)		
No	15/101			

* Knowledge and poverty were analyzed as continuous variables with average score values displayed in the table with maximum values as (average score/maximum score value). Knowledge and poverty interaction was taken.

** Adjusted odds ratios only displayed for variables that remained significant at alpha < 0.1

## Discussion

Rabies vaccine for humans and dogs have been in existence for over 100 years, and more than 30 countries have eliminated canine rabies through implementation of vaccination programs and laws enforcing responsible dog ownership [2,3]. Advances in canine rabies elimination have been steadily improving, and its elimination is a global priority [2,3]. This study assessed dog ownership and healthcare-seeking behaviors among residents of four communities in Cameroon. The results are meant to improve the implementation of canine rabies elimination programs in Cameroon and similarly impacted countries.

Here, we report the first rabies-focused knowledge, attitudes, and practices (KAP) survey conducted in Cameroon. Understanding of the dog population, dog bite injuries, and the health-seeking practices of community members can be used to improve rabies control measures. Almost 40% of the surveyed households owned dogs, representing a human to dog (H:D) ratio of 10.1:1, which was consistent with a previous ecologic study from North-West Cameroon (H:D of 8:1) [17]. Several studies have been conducted in Africa, showing similar human to dog ratios such as 10.8:1 in rural coastal Tanzania [18], and 11.1:1 in rural Southern Africa [19]. Previous studies have shown that dog populations are higher–relative to human populations–in rural areas as compared to urban areas [5,14,20–25]. Our findings further support this association, as the H:D ratio in these rural communities is 1.4 times more than that estimated for rural African communities (24.1:1). This finding reinforces the importance of stratified extrapolations of H:D ratios to obtain accurate dog population estimates over large geo-political areas.

Our study showed a significant association between dog ownership and wealth among these rural community members, where poor households were less likely to own dogs. This finding was also reported by Wallace and colleagues in Uganda, where they found that rates of dog ownership were low in impoverished rural areas [13]. Indeed, as reported previously by Wallace et al. [13] we identified a strong relationship between poverty and dog ownership, with impoverished communities claiming ownership of fewer dogs than wealthier communities. This further supports that simplistic extrapolations of dog populations based solely on human density may not be accurate, and other factors such as poverty should be considered when attempting to enumerate dog populations through mathematical methods. The findings here do support the suggestion that the impact of poverty on dog ownership may be a more common association than previously considered. While dog ownership was associated with wealth it was not associated with rabies knowledge.

A study from Uganda reported significant associations between poverty and dog vaccination coverage, with wealthier communities reporting higher coverage rates [13]. This association was not seen in the four rural Cameroon communities represented in this study. Similarly, knowledge of rabies had no bearing on dog vaccination. Dog rabies vaccine is often not available outside of government sponsored clinics in Cameroon. Therefore, the lack of association reported here may reflect a barrier in access to the vaccine that cannot necessarily be overcome by wealth or advanced rabies knowledge. This is not to say that if vaccine was available, knowledge and poverty would remain non-significant factors in dog vaccination, rather we propose that access is likely one of several limiting factors in dog vaccination rates. Cost was the main limiting factor for human PEP access among study participants; it should be expected that if access to canine vaccine is addressed, cost would surely be a limiting factor that would need to be considered. Future assessments should attempt to identify explanatory variables for this finding [26].

Of interest, the roaming profile observed for the dog population in this study was different than have been reported by others. While most studies have reported a high proportion of dogs roaming freely at all times (ranging from 31.4% to 78%) [13,14,27–31], our results demonstrated that only 1.4% of the dogs were allowed to always roam freely. While few dogs roam freely at all times in these communities, the proportion of dogs declared as confined at all times was consistent with prior observations (38%) [17]. It is known that dogs allowed to roam without supervision are more likely to be exposed to rabies virus due to contact with other dogs or wild mammals [31,32], helping on the persistence of domestic dog rabies, and implicating in high vaccination levels to prevent rabies [3, 33].

As previously discussed by Ratsitorahina et al. [34], interactions between owned and unowned dogs should be explored as an important aspect for rabies exposure within dog populations, and to non-reservoir populations (e.g. humans). Our survey showed that 95% of respondents provided partial care to their dogs, and more frequently with food, compared to a 10-fold decrease in partial care for community/non-owned dogs (only 9.1% of respondents). Furthermore, those 9.1% of people that cared for community dogs did not provide veterinary care. This survey only captured dog populations and vaccination coverage among the owned dog population. It is not possible to enumerate the community dog population with the methods used here. However, we can infer from this information that the community dog population may comprise at most 9% of the total dog population, and given that only central-point vaccination is practiced these dogs are unlikely to have a history of vaccination.

Awah-Ndukum et al. showed in their study that the costs associated with dog rabies vaccine in Cameroon are high [17], and even though national campaigns have been conducted, dog-owners are still responsible for part of the costs. This cost is thought to have contributed to low coverage rates during these campaigns [10,12]. However, a lack of access to qualified veterinary care cannot be ruled out in this population. It is likely that cost and access are contributing factors to the low utilization of veterinary care for owned dogs in this study, highlighting the importance of government services to provide essential veterinary services (i.e., rabies vaccination) at an affordable cost.

Attitudes and practices regarding bite events and possible rabies exposures were also addressed in this study (Table 6). Only 18% of bite victims assessed in this survey washed the wound, and even fewer respondents indicated that they would do so if bitten in the future (6%). These findings suggest that most respondents were unaware of this preventive measure, which can reduce the risk of successful rabies virus transmission by up to 40% [35]. Preventive measure of wound washing should be clearly emphasized during future rabies vaccination and education campaigns [10,36].

Over half of survey respondents indicated that they would seek medical care if they were bitten by a dog they did not know. However, in practice, among the 11 respondents with dog bites, only two sought care and only one received PEP. The individual reasons for not seeking medical care among bite victims were not investigated in this study, however cost was identified as a primary reason for not seeking medical care by all respondents and wealth was positively associated with the knowledge that healthcare should be sought if bitten. The same association was observed in regards to increased rabies knowledge. Most human deaths caused by rabies in developing countries are associated with failure to seek medical care [1,2,5], and the cost-barrier for dog rabies vaccination has also been reported in other Cameroonian communities [12] suggesting that poverty may be a barrier to accessing post-bite medical care. Further studies to explore barriers to medical care and associations between perceived risk and healthcare-seeking behaviors should be pursued.

Respondents with lower wealth scores were more likely to seek care from a traditional healer, as well as males and oldest individuals. However, it’s important to emphasize that the knowledge of rabies in the respondents’ community had a great impact on this association, due to individuals with more knowledge being more likely to seek medical care. Indeed, knowledge is an important factor for seeking care against rabies, but according to our analysis, knowledge is more helpful when people are wealthier. These findings suggest that improving knowledge about rabies prevention may not impact healthcare-seeking practices if the cost of medical care remains a perceived barrier.

There are important limitations to our findings. First, convenience sampling may not yield a truly representative sample of the entire population. Furthermore, questions focused on individuals’ attitudes could have resulted in inherent bias. Only the respondent was interviewed in relation to bite events, though not other members of the household. Consequently, if someone had been bitten, contracted rabies, and died, they were obviously excluded from this survey. Finally, the study population described here represents rural communities, expanding data collection to more communities that represent a continuum of poverty levels and urban status would improve extrapolation of this data to the entirety of Cameroon.

## Conclusion

In conclusion, our findings provide useful information focused on dog ownership and associated behaviors, population knowledge, attitudes and practices, and rabies exposures that could be applied to national rabies prevention efforts. This study identified numerous factors that may limit successful dog vaccination efforts in rural Cameroon, including a large proportion of free roaming dogs, access to the vaccine, and ability to pay for veterinary services. These multi-focal barriers will require a comprehensive approach that includes improved access to government services, educational campaigns, and dog population management. These same barriers could also limit human access to PEP, although cost may be much more important in regards to human vaccine decisions. Improving access to human PEP should address both costs and awareness, as this study indicates that despite awareness, if cost is a barrier then traditional medical approaches may still be utilized. The findings reported here are intended to improve rabies control practices in Cameroon and similar areas of Central Africa.

## Supporting information

S1 Appendix Data collection instrument (in English). (DOCX)
