# Supplementary material for: The influence of poverty and rabies knowledge on healthcare seeking behaviors and dog ownership, Cameroon
Source: PLoS One. 2018 Jun 21;13(6):e0197330. doi: 10.1371/journal.pone.0197330 (PMC6013156; doi:10.1371/journal.pone.0197330)
Supplement: S1 Appendix — (DOCX) [file pone.0197330.s001.docx]

**Appendix 1: Survey Questionnaire**

1. Interview Date:
2. Interviewer:
3. Consent obtained (Note: Form requests confirmation of adult age)
   1. Yes
   2. No
4. How old are you?
   1. 21-30
   2. 31-40
   3. 41-50
   4. 51-60
   5. 61-70
   6. Over 70
   7. Declined to answer
5. Gender
6. Male
7. Female
8. How many years of schooling have you completed?
9. None
10. 1-3
11. 4-6
12. 7-9
13. 10-12
14. Over 12
15. Declined to answer
16. How many people live in this household?
17. 1
18. 2
19. 3
20. 4
21. 5
22. 6
23. >6
24. Declined to answer
25. How many children below the age of 18 live in this household?
26. 1
27. 2
28. 3
29. 4
30. 5
31. 6
32. >6
33. Declined to answer
34. How many years have you lived in this community?
35. Less than 1 year
36. 1-5 years
37. 6-10 years
38. 11-15 years
39. 16-20 years
40. Over 20 years
41. Declined to answer
42. (Surveyor assistant - observe and describe construction of house).
43. Floor – cement / dirt / other:
44. Walls – cement / mud / nothing / other:
45. Roof – cement / thatched / other:
46. Windows – sealed / curtain / nothing / other:
47. Door – sealed / curtain / nothing / other:
48. What kind of livestock does your family own? How many head of each? Check all that apply.
49. None
50. Chickens, less than 10, 11-30, 31 or greater
51. Cattle, less than 10, 11-30, 31 or greater
52. Goats, less than 10, 11-30, 31 or greater
53. Sheep, less than 10, 11-30, 31 or greater
54. Other: (free response), less than 10, 11-30, 31 or greater
55. Declined to answer
56. Does your family currently own any dogs? If yes, how many? (**if answer is No, skip to 16**)
57. No
58. 1-2
59. 3-5
60. Greater than 5
61. Declined to answer
62. What best describes the amount of time that your dog(s) spends indoors?
63. Never
64. Infrequently
65. Occasionally
66. Frequently
67. Always
68. Declined to answer
69. What level of care do you provide for your dog(s)? Check all that apply.
70. None
71. Food
72. Water
73. Shelter
74. Veterinary Care
75. Other: (free response)
76. Declined to answer
77. Have any of your dog(s) been vaccinated against rabies?
78. Yes, all of them
79. Yes, some of them
80. No, none of them
81. I don’t know
82. Declined to answer
83. Does your family care for any dogs in the community? If yes, how many? (**if answer is No, skip to 18**)
84. No
85. 1-2
86. 3-5
87. Greater than 5
88. Declined to answer
89. What level of care do you provide for the community dog(s)? Check all that apply.
90. None
91. Food
92. Water
93. Shelter
94. Veterinary Care
95. Other: (free response)
96. Declined to answer
97. Have you been bitten by a dog in the past two years? (**if answer is No, skip to 26**)
98. No
99. Yes
100. Declined to answer
101. On how many separate occasions have you been bitten by a dog in the past two years?
102. 1-5
103. 6-10
104. 11-15
105. >16
106. Declined to answer
107. For the most recent event, where were you when you were bitten by the dog?
108. At home
109. Not at home, but within local community
110. Outside of local community
111. Declined to answer
112. For the most recent event, what were you doing when you were bitten the dog?
     1. At home, unprovoked attack by own dog
     2. At home, unprovoked attack by community dog
     3. Playing with, restraining or feeding the dog
     4. Playing with, restraining of feeding puppies of the (bitch) dog
     5. Visiting the dog’s home
     6. Walking in community, avoiding the dog
     7. Herding livestock, avoiding the dog
     8. Hunting wild animals, avoiding the dog
     9. Playing or recreating outdoors, avoiding the dog
     10. Other: (free response)
     11. Declined to answer
113. For the most recent event, where on your body were you bitten by the dog? Check all that apply.
114. Head/face
115. Torso/trunk
116. Hands/feet
117. Arm
118. Leg
119. Other: (free response)
120. Declined to answer
121. For the most recent event, what did you do when you were bitten by the dog? Check all that apply.
122. Nothing
123. Washed wound
124. Consulted with a traditional healer
125. Called a doctor
126. Actively sought medical treatment at a pharmacy, hospital, clinic or outpost
127. Received rabies post-exposure prophylaxis
128. Confined dog for observation
129. Submitted dog for disease testing
130. Killed dog
131. Other: (Free response)
132. Declined to answer
133. (Regarding option E of previous question) What was the amount of time between when you were bitten and when you sought medical treatment?
     1. < 1 day
     2. 1-3 days
     3. 4-6 days
     4. 1-2 weeks
     5. 3-4 weeks
     6. 5-8 weeks
     7. > 2 months
     8. Other: (free text)
     9. Option E was not part of answer to previous question
     10. Declined to answer
134. For the most recent event, how familiar were you with the dog that bit you?
135. Own (family) dog
136. Neighbor’s dog
137. Dog in community
138. Did not recognize dog
139. Declined to answer
140. (*if never been bitten by a dog*) What would you do if you were bitten by a dog that you know/own? Check all that apply.
141. Nothing
142. Wash wound
143. Consult with a traditional healer
144. Call a doctor
145. Actively seek medical treatment at a pharmacy, hospital, clinic or outpost
146. Receive rabies post-exposure prophylaxis
147. Confine dog for observation
148. Submit dog for disease testing
149. Kill dog
150. Other: (Free response)
151. Declined to answer
152. (*if never been bitten by a dog*) What would you do if you were bitten by a dog that you do not know/own? Check all that apply.
153. Nothing
154. Wash wound
155. Consult with a traditional healer
156. Call a doctor
157. Actively seek medical treatment at a pharmacy, hospital, clinic or outpost
158. Receive rabies post-exposure prophylaxis
159. Confine animal for observation
160. Submit animal for disease testing
161. Kill animal
162. Other: (Free response)
163. Declined to answer
164. If you saw a dog that looked sick, what would you do? Check all that apply.
165. Nothing
166. Call local authorities
167. Call a friend
168. Avoid the animal
169. Scare (shoo) animal away
170. Kill the animal
171. Submit the animal for disease testing
172. Other: (Free response)
173. Declined to answer
174. If you were bitten by a dog that looked sick, what would you do? Check all that apply.
175. Nothing
176. Wash wound
177. Consult with a traditional healer
178. Call a doctor
179. Actively seek medical treatment at a pharmacy, hospital, clinic or outpost
180. Receive rabies post-exposure prophylaxis
181. Confine animal for observation
182. Submit animal for disease testing
183. Kill animal
184. Other: (Free response)
185. Declined to answer
186. Have you or anyone you know had illness that was attributed to a pet/livestock animal bite in the past two years? (**if answer is No, skip to 32**)
187. Yes, myself
188. Yes, a family member or friend
189. Yes, someone I know in my community
190. Yes, multiple people that I know
191. No
192. Declined to answer
193. If yes to previous, what were the symptoms? If answer to previous question is ‘D’, describe for the most recent case? Check all that apply.
194. Skin rash/discoloration/ infection
195. Unusual bleeding (e.g. from nose/mouth)
196. Fever
197. Cough
198. Sneezing
199. Runny nose
200. Chest congestion
201. Muscle pain
202. Difficulty breathing
203. Headache
204. Convulsions
205. Altered mental state (dementia)
206. Unconsciousness/coma
207. Muscle weakness/paralysis
208. Vomiting or diarrhea or stomach cramps
209. Miscarriage/stillbirth
210. Death
211. Multiple persons
212. Other: (Free response)
213. Declined to answer
214. Have you been bitten by a wild animal in the past two years? (**if answer is No, skip to 40**)
215. No
216. Yes
217. Declined to answer
218. What kind of wild animal was it?
219. Jackal
220. Hyena
221. Mongoose
222. Honey badger
223. Monkey or other primate
224. Fox
225. Bat
226. Other:
227. I don’t know
228. Declined to answer
229. On how many separate occasions have you been bitten by a wild animal in the past two years?
230. 1-5
231. 6-10
232. 11-15
233. >16
234. I don’t know
235. Declined to answer
236. For the most recent event, where were you when you were bitten by the wild animal?
237. At home
238. Not at home, but within local community
239. Outside of local community
240. Declined to answer
241. For the most recent event, what were you doing when you were bitten by the wild animal?
242. In home, the animal entered home
243. Walking in community, avoiding the animal
244. Playing with, restraining or feeding the animal
245. Herding livestock, avoiding the animal
246. Hunting other animals
247. Hunting the animal
248. Playing or recreating outdoors, avoiding the animal
249. Other: (free response)
250. Declined to answer
251. For the most recent event, where on your body were you bitten by the wild animal? Check all that apply.
252. Head/face
253. Torso/trunk
254. Hands/feet
255. Arm
256. Leg
257. Other: (free response)
258. Declined to answer
259. For the most recent event, what did you do after you were bitten by the wild animal? Check all that apply.
260. Nothing
261. Washed wound
262. Consulted with a traditional healer
263. Called a doctor
264. Actively sought medical treatment at a pharmacy, hospital, clinic or outpost
265. Received rabies post-exposure prophylaxis
266. Confined animal for observation
267. Submitted animal for disease testing
268. Killed animal
269. Other: (Free response)
270. Declined to answer
271. (Regarding option E of previous question) What was the amount of time between when you were bitten and when you sought medical treatment?
     1. < 1 day
     2. 1-3 days
     3. 4-6 days
     4. 1-2 weeks
     5. 3-4 weeks
     6. 5-8 weeks
     7. > 2 months
     8. Other: (free text)
     9. Option E was not part of answer to previous question
     10. Declined to answer
272. If you saw a wild animal that looked sick, what would you do? Check all that apply.
273. Nothing
274. Call local authorities
275. Call a friend
276. Avoid the animal
277. Scare (shoo) animal away
278. Kill the animal
279. Submit the animal for disease testing
280. Other: (Free response)
281. Declined to answer
282. If you were bitten by a wild animal that looked sick, what would you do? Check all that apply.
283. Nothing
284. Wash wound
285. Consult with a traditional healer
286. Call a doctor
287. Actively seek medical treatment at a pharmacy, hospital, clinic or outpost
288. Receive rabies post-exposure prophylaxis
289. Confine animal for observation
290. Submit animal for disease testing
291. Kill animal
292. Other: (Free response)
293. Declined to answer
294. Have you or anyone you know had illness that was attributed to a wild animal bite in the past two years? (**if answer is No, skip to 44**)
295. Yes, myself
296. Yes, a family member or friend
297. Yes, someone I know in my community
298. Yes, multiple people that I know
299. No
300. Declined to answer
301. If yes to previous, what were the symptoms? If answer to previous question is ‘D’, describe for the most recent case? Check all that apply.
302. Skin rash/discoloration/ infection
303. Unusual bleeding (e.g. from nose/mouth)
304. Fever
305. Cough
306. Sneezing
307. Runny nose
308. Chest congestion
309. Muscle pain
310. Difficulty breathing
311. Headache
312. Convulsions
313. Altered mental state (dementia)
314. Unconsciousness/coma
315. Muscle weakness/paralysis
316. Vomiting or diarrhea or stomach cramps
317. Miscarriage/stillbirth
318. Death
319. Multiple persons
320. Other: (Free response)
321. Declined to answer
322. How much do you know about a disease called rabies?
323. Little to none
324. Basic understanding
325. Extensive knowledge
326. Declined to answer
327. How severe is the disease called rabies?
328. Mild
329. Somewhat severe
330. Very severe
331. I don’t know
332. Declined to answer
333. How do humans get rabies from an infected animal? Check all that apply.
334. Bite
335. Scratch
336. Observing the animal
337. Touching the animal
338. Contact with blood
339. Contact with saliva
340. Contact with urine/feces
341. Other: (free response)
342. I don’t know
343. Declined to answer
344. What animals can be infected with rabies? Check all that apply
345. Dogs
346. Cats
347. Livestock (Cattle, sheep, goats, etc.)
348. Poultry (Chickens, ducks, geese, etc.)
349. Horses
350. Jackals
351. Hyenas
352. Mongoose
353. Monkeys or other primate
354. Fox
355. Wild Birds
356. Bats
357. Rodents
358. Other: (free response)
359. I don’t know
360. Declined to answer
361. If you thought that you had an exposure to an animal with rabies, what would you do?
362. Nothing
363. Wash wound
364. Consult with a traditional healer
365. Call a doctor
366. Actively seek medical treatment at a pharmacy, hospital, clinic or outpost
367. Receive rabies post-exposure prophylaxis
368. Confine animal for observation
369. Submit animal for disease testing
370. Kill animal
371. Other: (Free response)
372. Declined to answer
373. How far away is the closest pharmacy?
     1. <1km
     2. 1-5km
     3. 6-10km
     4. 11-20km
     5. 21-30km
     6. >30km
     7. I don’t know
     8. Declined to answer
374. How far away is the closest medical clinic?
375. <1km
376. 1-5km
377. 6-10km
378. 11-20km
379. 21-30km
380. >30km
381. I don’t know
382. Declined to answer
383. How far away is the closest hospital?
384. <1km
385. 1-5km
386. 6-10km
387. 11-20km
388. 21-30km
389. >30km
390. I don’t know
391. Declined to answer
392. How far away is the closest traditional healer?
393. <1km
394. 1-5km
395. 6-10km
396. 11-20km
397. 21-30km
398. >30km
399. I don’t know
400. Declined to answer
401. What obstacles do you think exist for getting medical treatment in your community? Check all that apply.
402. Lack of facilities to provide treatment
403. Lack of trained personnel at facilities to provide treatment
404. Lack of medicines at facilities for treatment
405. No means of transportation
406. No money to pay for treatment
407. Can’t miss work
408. Other: (free text)
409. I don’t know
410. Declined to answer

***Those are all the questions I have for you. Thank you very much for your time and cooperation.***
